# Safety and Efficacy of a Fixed-Dose Combination of Trypsin, Bromelain, Rutoside, and Diclofenac in Wound Management: A Randomized Clinical Trial

**DOI:** 10.7759/cureus.88228

**Published:** 2025-07-18

**Authors:** Bhupesh Dewan, Siddheshwar Shinde, Nisha Motwani

**Affiliations:** 1 Medical Services, Zuventus Healthcare Limited, Mumbai, IND

**Keywords:** bromelain, diclofenac, fixed-dose combination, orthopedic surgery, pain, proteolytic enzymes, rutin, trypsin, wound care

## Abstract

Background

One of the most significant postoperative challenges faced by patients is the occurrence of acute episodes of pain and inflammation, often accompanied by the development of edema, particularly in the case of large wounds. Consequently, any delay in the healing process of such wounds can exert a substantial impact on recovery. This delay can potentially be mitigated by addressing the moisture imbalance at the wound site using proteases, while concurrently minimizing pain and inflammation through the use of appropriate analgesic and anti-inflammatory preparations. The objective of this study was to evaluate the safety and efficacy of Tibrolin® D, a fixed-dose combination comprising trypsin, bromelain, rutoside, and diclofenac tablets, in alleviating pain intensity and enhancing wound healing in patients experiencing acute pain resulting from uncontaminated minor orthopedic surgical procedures or wounds.

Method

An open-label, multicenter, controlled, comparative Phase IV clinical study was conducted on 200 patients who underwent elective, clean, and uncontaminated minor orthopedic surgery. These patients were randomized (1:1) to receive either Tibrolin® D (a combination of trypsin 48 mg, bromelain 90 mg, rutoside 100 mg, and diclofenac 50 mg) or Voveran® 50 GE (diclofenac 50 mg) as one tablet orally, thrice a day postoperatively for seven days. The primary outcome was the proportion of patients reporting adverse events. Secondary outcomes included a total wound symptom score, evaluated based on symptoms after minor orthopedic surgery using a four-point Likert scale, and pain intensity assessed by the Numerical Pain Rating Scale.

Results

No adverse events were observed in this study. Both groups demonstrated statistically significant improvement (p < 0.001) in all assessed wound symptoms: erythema, edema, discharge, induration, local irritation, tenderness, and pain. By Day 7, total wound symptom scores significantly decreased in both the Tibrolin® D group (12.84 ± 2.89 to 2.24 ± 2.08) and the diclofenac group (12.86 ± 3.29 to 2.66 ± 2.28) (p < 0.001), with no significant difference between groups (p = 0.318). Pain intensity also decreased significantly in both groups by Day 7 (p < 0.001), with no significant difference in mean change between groups (p = 0.412). Notably, 91% of patients and 96% of investigators rated Tibrolin® D as good to excellent in relieving wound symptoms, comparable to ratings for diclofenac (88% and 96%, respectively).

Conclusion

Tibrolin® D was as well tolerated as diclofenac, indicating a favorable safety profile. The findings also suggest that Tibrolin® D improves wound healing symptoms and reduces pain intensity following minor orthopedic surgeries, demonstrating efficacy comparable to diclofenac.

## Introduction

Impaired tissue repair affects over 10.5 million individuals worldwide each year, representing a significant medical challenge that contributes to increased morbidity, mortality, and reduced quality of life [1]. Acute wounds, particularly those resulting from orthopedic surgical procedures, require optimized management to prevent progression to chronic, non-healing wounds, which can increase healthcare costs and patient burden [2].

Effective wound healing depends on timely intervention to restore tissue perfusion and resolve inflammation, both of which are critical for successful tissue repair [3]. Non-healing wounds often result from prolonged inflammation, where an imbalance between proteases and their inhibitors disrupts extracellular matrix deposition and degradation [4]. This imbalance, along with impaired tissue perfusion, compromises oxygen and nutrient delivery, ultimately hindering the repair process.

Wound healing unfolds in three phases, primary healing, granulation, and scar formation, each dependent on adequate blood supply, tissue perfusion, and biochemical factors [5]. Injury triggers vascular permeability, leading to plasma protein efflux and fluid retention within the first 72 hours. Poor tissue perfusion exacerbates fluid accumulation, disrupts collagen organization, impairs lymphatic circulation, and promotes bacterial growth, thereby delaying healing [3,6,7].

Current wound management strategies often fall short in addressing the multifaceted pathophysiological mechanisms underlying tissue damage and healing. Systemic proteolytic enzymes such as trypsin, bromelain, and rutoside have demonstrated potential in regulating tissue fluid balance, enhancing microcirculation, and improving tissue perfusion [8]. Trypsin binds to α1-antitrypsin, enhancing plasmin bioavailability for fibrinolysis, which clears fibrin clots and restores microcirculation, thereby supporting tissue perfusion and remodeling. Bromelain, through cyclooxygenase-2 (COX-2) and prostaglandin E2 inhibition, reduces inflammation and modulates pain via bradykinin pathways [9,10]. Rutoside improves capillary strength, reduces vascular permeability, and enhances perfusion by neutralizing oxidative stress and improving venous return [11]. This combination has shown comparable efficacy to diclofenac [12,13] and ibuprofen [14].

Diclofenac, a widely used nonsteroidal anti-inflammatory drug (NSAID), inhibits COX-mediated prostaglandin synthesis to provide analgesic and anti-inflammatory effects [15,16]. However, the combination of proteolytic enzymes with diclofenac has been minimally explored in the context of acute post-orthopedic surgical wound care. This randomized clinical trial was therefore designed to evaluate the safety and efficacy of a fixed-dose combination of trypsin, bromelain, rutoside, and diclofenac in improving wound healing and pain management in patients with acute, uncontaminated minor orthopedic surgical wounds.

## Materials and methods

Study design and ethics

This was an open-label, prospective, multicentric, randomized, controlled, comparative, Phase-IV clinical study conducted at four geographically distributed sites across India. The study was carried out in accordance with the protocol and the requirements of the New Drugs and Clinical Trials Rules 2019, Ethical Guidelines for Biomedical Research on Human Participants by the Indian Council of Medical Research (2017), International Council for Harmonisation Guidelines E6 (R2) for Good Clinical Practice, and the Declaration of Helsinki (Brazil, October 2013). The study was initiated after receiving approval from the Drug Controller General of India (DCGI) and the respective institutional ethics committees at each of the study centers. The trial was registered with the Clinical Trial Registry of India (CTRI/2021/03/032053).

Eligibility criteria

The study enrolled 200 patients of both sexes, aged between 18 and 65 years, who underwent elective, clean, and uncontaminated minor orthopedic surgery. All patients provided written informed consent to participate in the study. Patients were excluded if they had a history of hypersensitivity to any of the ingredients in the formulation; had hepatocellular insufficiency, hepatic failure, or active liver disease; had severe renal impairment; had a hereditary coagulation disorder; or were pregnant or breastfeeding.

Treatments

Eligible patients were randomized (1:1) to receive either the test product, Tibrolin® D (a combination of trypsin 48 mg, bromelain 90 mg, rutoside 100 mg, and diclofenac 50 mg tablet) marketed by Zuventus Healthcare Limited, or the comparator product, Voveran® 50 GE (diclofenac 50 mg tablet) marketed by Novartis India Limited. Each patient took one tablet of the assigned product orally, three times a day, for 7 days following surgery. Patients were provided with a diary containing a predefined schedule to record their intake of the assigned treatment. At each follow-up visit, treatment compliance was assessed using a questionnaire and the patient diary.

Study procedure

All patients who provided duly signed informed consent and met the inclusion criteria were enrolled in the study. The study treatment was assigned according to the randomization schedule. The treatment lasted for 7 days and required 4 visits: screening (baseline), day 3, day 5, and day 7. The patients’ vital signs (blood pressure, heart rate, temperature, and respiration rate) were monitored, and adverse events were recorded at each follow-up visit for safety evaluation. Serum creatinine, blood urea nitrogen, total bilirubin, alanine aminotransferase, alanine transaminase, complete blood count, erythrocyte sedimentation rate, and C-reactive protein were checked at baseline and on day 7. Using patients' clinical signs and symptoms, improvement in wound healing and pain intensity was assessed by orthopedic surgeons on days 3, 5, and 7. At the end of the study, global assessment of treatment tolerability was evaluated based on both the patients’ and investigators’ responses. All patients received a patient diary containing information on the 7-day medicine administration regimen to monitor treatment compliance. Treatment compliance was evaluated at each follow-up visit through both the questionnaire and patient diary.

Study assessment

The primary outcome of the study was the proportion of patients reporting adverse events. Safety was assessed throughout the study based on observed adverse events and their relationship to the investigational drug, as determined by the WHO-Uppsala Monitoring Centre scale.

Secondary outcomes included changes in the total wound symptom score, which covered symptoms such as erythema, edema, discharge, induration, local irritation, and tenderness. These symptoms were scored at baseline and on days 3, 5, and 7 using a four-point Likert scale (0 = absent, 1 = mild, 2 = moderate, 3 = severe). The total score was calculated by summing individual symptom scores, with a maximum of 18 points [8]. The reduction in total score was expressed as a percentage. Pain intensity was assessed using a validated 11-point Numerical Pain Rating Scale (NPRS), ranging from 0 (no pain) to 10 (worst possible pain) [17]. Changes in pain scores from baseline were recorded at each follow-up. On day 7, both investigators and patients provided a global assessment of treatment tolerability using a five-point Likert scale (1 = excellent, 2 = good, 3 = average, 4 = no response, 5 = poor).

Statistical analysis

Based on previous literature, a comparable reduction in pain intensity was observed with proteolytic enzymes (40.6%) and diclofenac (40.9%) on the 11-point NPRS [18]. To detect at least a 60% improvement with the proteolytic enzyme-diclofenac combination, a sample size of 174 patients (87 per group) was calculated to achieve 80% statistical power at a 5% level of significance. Considering a 10% dropout rate, 200 patients (100 per group) were enrolled.

Baseline characteristics were reported as mean ± SD or number (%). These characteristics were analyzed using an unpaired t-test and chi-square test. Adverse events were presented as number (%). Wound symptom scores and pain intensity scores were presented as mean ± SD for each visit and analyzed using repeated measures ANOVA. Between-group comparisons for reductions in symptom and pain scores were performed using unpaired t-tests. A p-value of <0.05 was considered statistically significant. Global assessment of treatment tolerability, based on both investigator and patient responses, was presented as the percentage of patients corresponding to each response category.

## Results

Baseline characteristics

**Figure 1:**
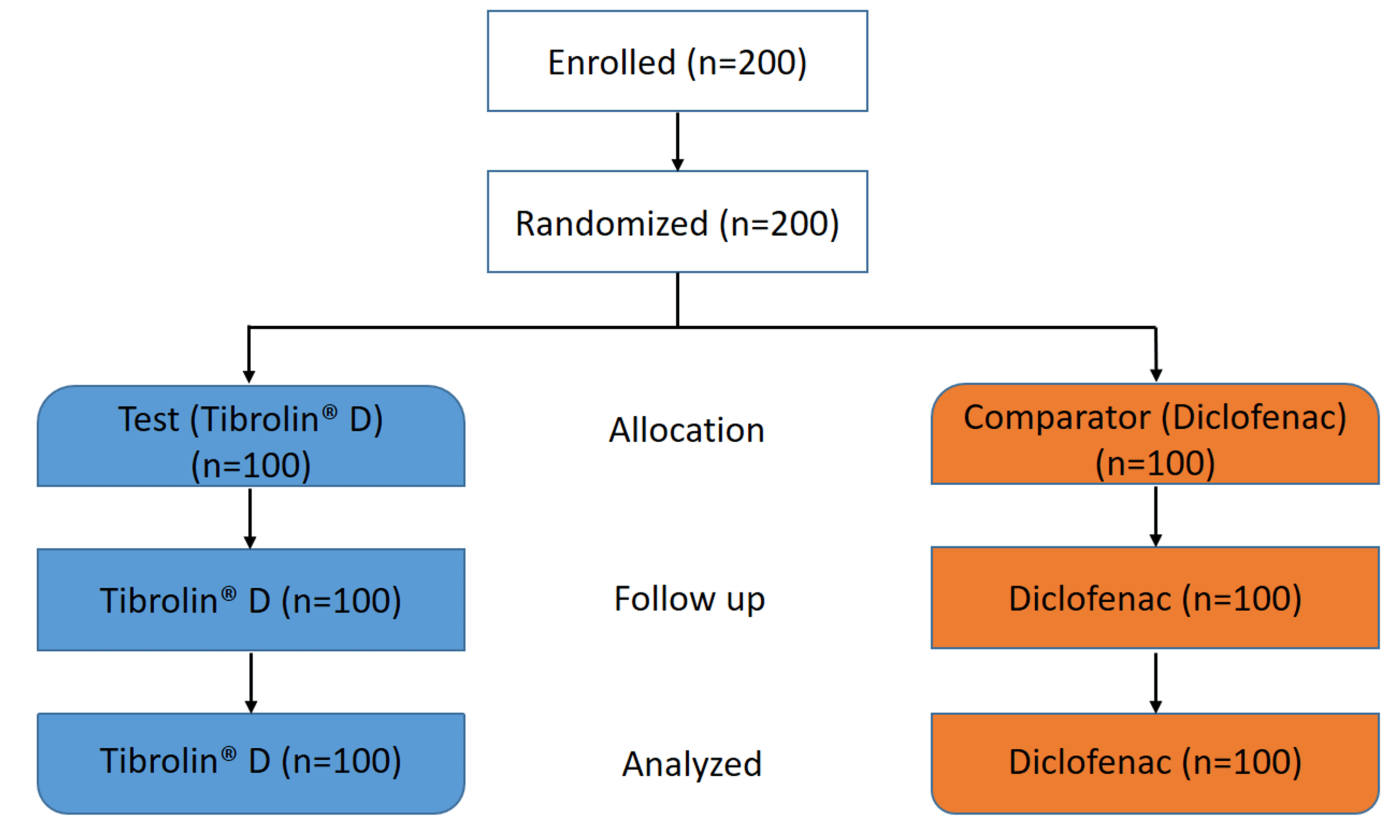
Disposition of patients.

A total of 200 eligible patients who had undergone elective, clean, and uncontaminated minor orthopedic surgeries were enrolled and randomized in a 1:1 ratio to receive either Tibrolin D® or Voveran® 50 GE during the trial conducted from April 2021 to September 2021. The CONSORT flow diagram of the study is presented in Figure 1. The mean age of the enrolled patients was 38.4 years. None of the patients dropped out or discontinued the study. The most commonly administered concomitant medications in both groups were antibiotics, vitamins and minerals, and acid suppressants/prokinetics post-surgery. Overall, the demographic and baseline characteristics were comparable between the treatment groups. Detailed baseline demographics are summarized in Table 1.

**Table 1:** Patient demographic data and baseline characteristics. Data are presented as mean ± standard deviation unless otherwise specified as n (%). * t-value (test statistic) from the unpaired t-test. ** χ²-value (test statistic) from the Pearson chi-square test. ^#^ Data analyzed using the unpaired t-test. ^##^ Data analyzed using the Pearson chi-square test. SBP: Systolic Blood Pressure; DBP: Diastolic Blood Pressure.

Characteristics	Tibrolin® D	Diclofenac	Test statistic*	p-value#
Age (years)	38.74 ± 12.92	38.06 ± 13.07	-0.37	0.7118
Gender, n (%)				
Male	76 (76.00%)	74 (74.00%)	0.1067**	0.7440##
Female	24 (24.00%)	26 (26.00%)		
Weight (kg)	66.95 ± 10.70	67.03 ± 9.98	0.0506	0.9597
Height (cm)	164.86 ± 8.80	164.20 ± 9.77	0.6929	0.6143
SBP (mmHg)	122.44 ± 5.35	122.10 ± 5.21	-0.4553	0.6494
DBP (mmHg)	80.99 ± 4.87	81.18 ± 5.44	0.2601	0.7951
Pulse rate (beats/min)	83.21 ± 6.48	83.21 ± 6.48	-0.4268	0.67
Pain intensity, n (%)				
Mild	6 (6.00%)	5 (5.00%)	0.8684**	0.6480##
Moderate	28 (28.00%)	34 (34.00%)		
Severe	66 (66.00%)	61 (61.00%)		
Types of surgeries, n (%)				
Upper limb fractures and injuries	37 (37.00%)	42 (42.00%)	1.2881**	0.8630##
Spinal surgery	3 (3.00%)	2 (2.00%)		
Lower limb fractures and injuries	35 (35.00%)	36 (36.00%)		
General surgery	19 (19.00%)	14 (14.00%)		
Implant removal	6 (6.00%)	6 (6.00%)		

Safety assessment

No clinically significant changes were observed in vital signs or laboratory investigations following the completion of either treatment period. Furthermore, no adverse events were reported during the study.

Efficacy assessment

Reduction in the Severity of Wound Symptoms

The reduction in wound symptom scores for erythema, edema, wound discharge, induration, local irritation, and tenderness at each follow-up visit, when compared to the baseline visit, was significantly higher (p<0.001) in the Tibrolin® D group. Additionally, a significant reduction in wound discharge was observed on Day 3 between the two groups (p=0.0171), suggesting a possible improvement in wound drainage with the use of systemic oral enzymes. The scores for each wound symptom are provided in Table 2.

**Table 2:** Change in wound symptom score from baseline to Days 3, 5, and 7. Repeated measures analysis of variance (ANOVA-RM) was used to compare changes in wound symptom scores between Days 0, 3, 5, and 7. *Between-group comparisons at Days 3, 5, and 7 were analyzed using an unpaired t-test. ANOVA-RM: Repeated Measures Analysis of Variance; DBP: Diastolic Blood Pressure.

Wound symptoms	Tibrolin^®^ D (mean ± SD)	Diclofenac (mean ± SD)	Between-group difference*	
			Mean difference (95%CI)	p-value
Erythema				
Baseline	2.49 ± 0.64	2.45 ± 0.81	-	-
Day 3	1.83 ± 0.68	1.84 ± 0.77	-0.05 (-0.19, 0.09)	0.4746
Day 5	1.13 ± 0.66	1.19 ± 0.66	-0.1 (-0.28, 0.08)	0.2740
Day 7	0.44 ± 0.56	0.57 ± 0.61	-0.17 (-0.39, 0.04)	0.1200
p-value (ANOVA-RM)	<0.0001	<0.0001	-	-
Edema				
Baseline	2.35± 0.66	2.47 ± 0.59	-	-
Day 3	1.82 ± 0.71	1.96 ± 0.74	-0.02 (-0.17, 0.13)	0.7904
Day 5	1.23 ± 0.76	1.25 ± 0.67	0.1 (-0.08, 0.28)	0.2782
Day 7	0.51 ± 0.58	0.61 ± 0.62	0.02 (-0.18, 0.22)	0.8443
p-value (ANOVA-RM)	<0.0001	<0.0001	-	-
Discharge symptom				
Baseline	1.61 ± 1.12	1.61 ±1.16	-	-
Day 3	0.97 ± 0.95	1.16 ± 1.02	-0.19 (-0.35, -0.03)	0.0171
Day 5	0.66 ± 0.81	0.72 ± 0.83	-0.06 (-0.27, 0.15)	0.5708
Day 7	0.28 ± 0.47	0.34 ± 0.52	-0.06 (-0.32, 0.2)	0.6474
p-value (ANOVA-RM)	<0.0001	<0.0001	-	-
Induration symptoms				
Baseline	2.16 ± 0.73	2.22 ± 0.68	-	-
Day 3	1.51 ± 0.76	1.57 ± 0.72	0 (-0.18, 0.18)	1.0000
Day 5	0.94 ± 0.68	1.01 ± 0.73	-0.01 (-0.22, 0.20)	0.9264
Day 7	0.43 ± 0.54	0.47 ± 0.61	0.02 (-0.20, 0.24)	0.8597
p-value (ANOVA-RM)	<0.0001	<0.0001	-	-
Tenderness				
Baseline	2.01 ± 0.64	2.06 ± 0.68	-	-
Day 3	1.35 ± 0.66	1.48 ± 0.76	-0.08 (-0.25, 0.09)	0.3585
Day 5	0.9 ± 0.64	0.91 ± 0.60	0.04 (-0.15, 0.23)	0.6795
Day 7	0.25 ± 0.44	0.36 ± 0.48	-0.06 (-0.24, 0.12)	0.5186
p-value (ANOVA-RM)	<0.0001	<0.0001	-	-
Local irritation				
Baseline	2.22 ± 0.61	2.05 ± 0.80	-	-
Day 3	1.56 ± 0.64	1.39 ± 0.75	0 (-0.16, 0.16)	1.0000
Day 5	0.94 ± 0.63	0.81 ± 0.72	-0.04 (-0.24, 0.16)	0.6945
Day 7	0.33 ± 0.51	0.31 ± 0.53	-0.15 (-0.35, 0.05)	0.1353
p-value (ANOVA-RM)	<0.0001	<0.0001	-	-
Total wound symptom				
Baseline	12.84 ± 2.89	12.86 ± 3.29	-	-
Day 3	9.04 ± 2.96	9.40 ± 3.39	-0.34 (-0.91, 0.23)	0.2385
Day 5	5.8 ± 2.92	5.89 ± 2.97	-0.07 (-0.84, 0.70)	0.8584
Day 7	2.24 ± 2.08	2.66 ± 2.28	-0.4 (-1.19, 0.39)	0.3181
p-value (ANOVA-RM)	<0.0001	<0.0001	-	-

The total wound symptom score significantly decreased in both the Tibrolin® D group (from 12.84 ± 2.89 to 2.24 ± 2.08) and the diclofenac group (from 12.86 ± 3.29 to 2.66 ± 2.28) by Day 7 (p<0.001), with no statistically significant difference between the groups (p=0.3181).

**Figure 2:**
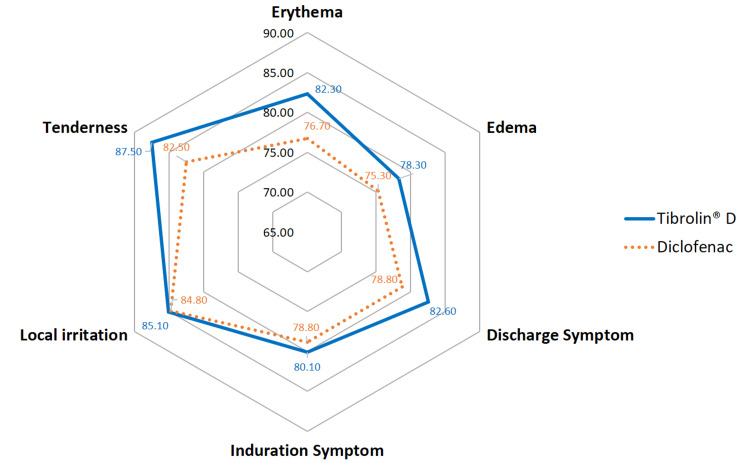
Reduction in wound symptom scores (%) from baseline to Day 7.

The radar plot (Figure 2) illustrates the percentage change in wound symptom scores from baseline to Day 7 for both treatment groups. Tibrolin® D showed a greater percent reduction in symptoms such as discharge, erythema, and tenderness compared to diclofenac. Overall, Tibrolin® D demonstrated better efficacy in symptom resolution over the 7-day treatment period.

Reduction in Pain Intensity

In the Tibrolin® D group, pain intensity significantly decreased from 6.6 ± 1.90 to 1.2 ± 0.90 (p < 0.001), and in the diclofenac group from 6.50 ± 1.74 to 1.31 ± 1.07 (p < 0.001) by Day 7, with no significant difference in the mean change between groups (p = 0.4125). An 82% relative reduction in pain intensity was observed with Tibrolin® D, compared to a 79.8% reduction with diclofenac alone. The results are summarized in Table 3.

**Table 3:** Change in pain intensity score from baseline to days 3, 5, and 7. Repeated Measures Analysis of Variance (ANOVA-RM) was used to compare changes in pain scores across baseline, day 3, day 5, and day 7. * Between-group comparisons at days 3, 5, and 7 were analyzed using an unpaired t-test.

Time Point	Tibrolin® D (mean ± SD)	Diclofenac (mean ± SD)	Between-group difference*	
			Mean difference (95%CI)	p-value
Baseline	6.60 ± 1.90	6.50 ± 1.74	–	–
Day 3	4.56 ± 1.42	4.59 ± 1.43	-0.13 (-0.44; 0.18)	0.4125
Day 5	2.88 ± 1.14	2.97 ± 1.23	-0.19 (-0.61; 0.23)	0.3724
Day 7	1.20 ± 0.90	1.31 ± 1.07	-0.21 (-0.71; 0.29)	0.4118
p-value (ANOVA-RM)	<0.0001	<0.0001	–	–

Global Assessment of Patients’ and Investigators’ Response to Study Treatment

**Figure 3:**
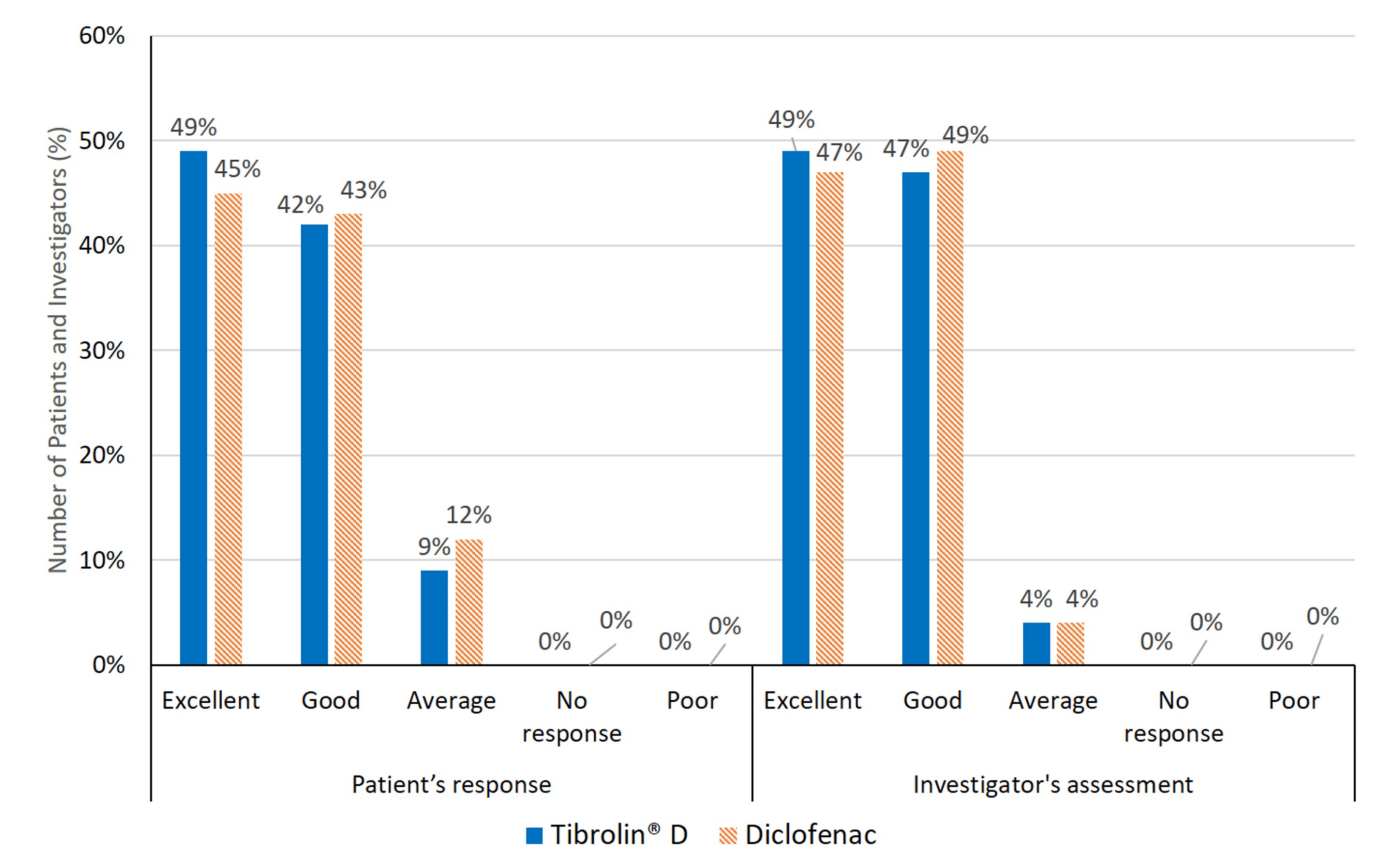
Global assessment of treatment tolerability.

There were no reports of dose adjustments or study modifications due to intolerance or adverse effects related to the study drug. Approximately 50% of patients and clinical investigators rated the study medication as excellent, while more than 40% of the remaining patients and investigators recorded a good response to the study treatment. These responses are graphically represented in Figure 3.

## Discussion

The pain associated with orthopedic surgical procedures primarily arises from tissue trauma, leading to local edema and inflammation at the wound site. Effective control of this inflammation is essential to reduce pain, accelerate wound healing, and enhance post-operative recovery. Several clinical trials have explored the efficacy and safety of oral proteolytic enzymes, particularly trypsin, bromelain, and rutoside, in post-surgical wound management [8,19,20-22]. These enzymes exhibit anti-inflammatory, fibrinolytic, and antioxidant properties [23], making them valuable in managing post-surgical symptoms.

The addition of diclofenac to the proteolytic enzymes provides a synergistic therapeutic benefit in managing inflammatory wound symptoms [24,25]. While the enzymes facilitate proteolytic debridement, improve microcirculation, and reduce oxidative stress, diclofenac offers potent COX-mediated anti-inflammatory and analgesic effects. This multimodal approach, combining trypsin, bromelain, rutoside, and diclofenac, enhances the resolution of wound symptoms and reduces pain intensity compared to diclofenac alone, as evaluated by Mungantiwar A et al. [26].

In the present study, the combination of proteolytic enzymes and diclofenac was well tolerated, with no reported adverse events. Divided, short-term dosing of diclofenac helped minimize GI side effects, aligning with earlier studies that support the safety of enzyme-NSAID-based oral therapies [27,28]. Both treatment groups showed early and marked reductions in pain intensity and wound symptoms. However, a comparative analysis using a radar chart indicated that Tibrolin® D demonstrated better improvement in wound-related symptoms. The broader spread of data points in the Tibrolin® D group reflects greater symptom resolution, suggesting a therapeutic advantage for this combination in the short-term management of minor orthopedic surgical wounds. These observed benefits may be attributed to enhanced local microcirculation, which promotes tissue repair. Mungantiwar A et al. [26] reported that the trypsin, bromelain, rutoside, and diclofenac combination significantly improved necrotic tissue reduction, exudate clearance, wound color, and epithelialization compared to diclofenac alone (p<0.05) in patients with post-minor surgical wounds.

Jayachandran S and Khobre P et al. [27] evaluated the efficacy of a combination therapy consisting of oral bromelain, trypsin, rutoside, and diclofenac in patients with temporomandibular joint (TMJ) osteoarthritis and found it to be more effective than diclofenac alone (p<0.01). Similarly, Gupta P et al. [28] assessed this combination for managing internal derangement of the TMJ and reported a highly significant (p<0.001) improvement in pain and function compared to diclofenac. The combination demonstrated greater efficacy, particularly in oral surgical procedures where localized inflammation and soft tissue swelling contribute to pain. The findings from a systematic review by Shekhar A et al. [29] further support the superior efficacy of this enzyme-diclofenac-based combination over diclofenac monotherapy. Chandra BR et al. [30] reported that this combination was more efficacious than the aceclofenac-paracetamol-serratiopeptidase combination in reducing postoperative complications following mandibular third molar surgery.

Together, these agents act synergistically to mitigate inflammation, relieve pressure from edematous tissues, and support the healing process. Their role in modulating cytokine activity and enhancing tissue perfusion makes them a rational therapeutic option in the post-operative setting, offering not only symptom control but also improved wound healing outcomes. However, larger, well-designed clinical trials are warranted to further validate the therapeutic efficacy and broaden the clinical application of this combination in post-surgical wound management.

## Conclusions

The fixed-dose combination Tibrolin® D (trypsin, bromelain, rutoside, and diclofenac) demonstrates significant therapeutic potential in the management of elective, clean, and uncontaminated minor orthopedic surgical wounds. By targeting multiple inflammatory pathways, this multimodal regimen effectively reduces pain, minimizes edema, and accelerates wound healing. Clinical evidence suggests superior efficacy over diclofenac alone, particularly in cases with substantial soft tissue inflammation. The combination was well tolerated, with no adverse events reported. These findings support the clinical use of Tibrolin® D as a safe and effective option for post-operative wound management in orthopedic settings.
